# Effects of the Saliva of Patients Undergoing Orthodontic Treatment with Invisalign and Brackets on Human Gingival Fibroblasts and Oral Epithelial Cells

**DOI:** 10.3390/jcm12237440

**Published:** 2023-11-30

**Authors:** Michael Nemec, Christian Behm, Marcus Sedlak, Hemma Nemec-Neuner, Phuong Quynh Nguyen, Erwin Jonke, Oleh Andrukhov

**Affiliations:** 1 Clinical Division of Orthodontics, University Clinic of Dentistry, Medical University of Vienna, 1090 Vienna, Austria; michael.nemec@meduniwien.ac.at (M.N.); hemma.nemec-neuner@meduniwien.ac.at (H.N.-N.); erwin.jonke@meduniwien.ac.at (E.J.); 2 Competence Center for Periodontal Research, University Clinic of Dentistry, Medical University of Vienna, 1090 Vienna, Austria; christian.behm@meduniwien.ac.at (C.B.); marcus_sedlak@aon.at (M.S.); phuong.nguyen@meduniwien.ac.at (P.Q.N.)

**Keywords:** orthodontic treatment, saliva, epithelial cells, human gingival fibroblasts, inflammatory mediators, epithelial barrier

## Abstract

The transient worsening of oral health sometimes accompanies orthodontic treatment (OT), and the extent of this effect might depend on whether the patients are treated with traditional brackets or clear aligners. Saliva is an important tool for monitoring oral health and influences the functional properties of various oral cells. This study aimed to compare the effects of saliva from patients undergoing OT with Invisalign aligners and brackets on human gingival fibroblasts and oral epithelial cells in vitro. Unstimulated saliva was collected from 15 patients treated with Invisalign and 16 patients treated with brackets before and 3 and 6 months after therapy began. The saliva was used to stimulate primary human gingival fibroblasts and the oral epithelial Ca9-22 cell line, and the resulting cell response was investigated. Saliva did not exhibit any toxic effect on investigated cells, as shown by the proliferation/viability assay with the MTT method. In human gingival fibroblasts, saliva increased gene expression of various proinflammatory mediators, such as interleukin (IL)-6, IL-8, and monocyte chemoattractant protein-1, assessed by qPCR. In epithelial cells, saliva increased the production of IL-8 measured by ELISA and decreased gene expression of various proteins involved in the barrier function. During the therapy, the saliva-induced production of IL-8 tended to be decreased, and the saliva-induced decrease in the expression of barrier protein was partially improved. No difference between aligners and brackets was observed in either cell type. Saliva affects the functional properties of oral cells, but this effect is not influenced by the type of OT.

## 1. Introduction

Orthodontic aligners have become increasingly popular in clinical use due to advantages in visual appearance and convenience and have been introduced as an esthetic alternative to multibracket appliances [1]. Aligners represent an orthodontic treatment (OT) modality providing a variable number of vacuum-formed removable plastic trays that gradually move teeth in the desired position. Aligners should be worn around the clock but not during food intake and oral hygiene procedures. Most treatment regimens require a sequential change to the next set of aligners after two weeks. Depending on the complexity of the patient’s malocclusion, the dentist must decide upon the number of aligners [2]. Thus, treatment duration can range from several months to years, a time when oral tissues are closely exposed to the plastic aligner material [3]. The world market leader in the field of orthodontic aligners is Align Technology (San Jose, CA, USA), and its product Invisalign^®^ has more than 15 million aligner treatments (https://www.invisalign-professional.de/ accessed on 7 September 2023).

Orthodontic treatment, especially bracket treatment, can lead to enhanced plaque accumulation and impaired possibilities of plaque removal due to restricted accessibility for oral hygiene procedures [4]. Comparative studies have shown that removable aligners can lead to better oral hygiene and periodontal health outcomes than fixed bracket treatment [4,5]. A recent systematic review and meta-analysis of randomized clinical trials and cohort studies found a lower plaque index for patients with clear aligners than those with fixed brackets [6]. However, the level of evidence still seems to be insufficient to conclude the superiority of aligners [6]. A systematic review of four systematic reviews also confirmed a slight tendency for clear aligners to be associated with slightly better periodontal conditions [7]. However, the differences in plaque index, gingival index, and bleeding on probing were classified as negligible in clinical settings, and therefore, clear aligners and fixed appliances were concluded to have a comparable impact on periodontal health status [7].

Saliva is a promising modality for monitoring oral health and diseases [8,9,10]. Saliva is composed mainly of water and contains different minerals, proteins, peptides, bacteria, and bacterial components. Periodontal disease is usually associated with increased salivary content of various inflammatory proteins [11]. The increased salivary levels of macrophage inflammatory protein (MIP)-1α and prostaglandin E2 (PGE2) but not interleukin (IL)-1β, IL-6, and IL-8 are reported in gingivitis, an initial stage of periodontitis [12]. A recent systematic review and meta-analysis suggests MIP-1α IL-1β, IL-6, and MMP-8 as highly sensitive salivary markers of periodontitis [13]. Thus, gingival inflammation is generally related to the increased content of the inflammatory proteins in saliva. Several studies showed that saliva can also induce the inflammatory response in different host cells, including mouse-derived macrophages, human gingival fibroblasts, and periodontal ligament cells [14,15]. A recent study found that saliva of both periodontitis patients and healthy controls increases the production of IL-6 and IL-8 and transepithelial electric resistance of oral epithelial cells by an approximately similar extent [16]. However, to date, it has yet to be discovered if and how OT influences the ability of saliva to stimulate the inflammatory response in various host cells.

The primary aim of this longitudinal study was to investigate how the ability of saliva to stimulate human oral epithelial cells and gingival fibroblasts changes during orthodontic therapy. The secondary aim was to compare how these parameters are influenced by different OT modalities, namely fixed brackets and clear aligners. These data are important to understand the potential effect of different types of OT more comprehensively.

## 2. Materials and Methods

### 2.1. Ethical Aspects

The Ethics Committee of the Medical University of Vienna (Vienna, Austria) has approved the protocols for the clinical examination and saliva collection from orthodontic patients (protocol number 1288/2019) and the isolation of human gingival fibroblasts (protocol number 1079/2019). Written informed consent was obtained from each patient before any study-related procedure. The underlying study was conducted in compliance with the Declaration of Helsinki and the “Good Scientific Practice” guidelines of the Medical University of Vienna. 

### 2.2. Study Participants

This pilot study included 15 patients treated with a buccal multibracket appliance of a 0.022-inch slot size (Empower^®^, American Orthodontics, Weil am Rhein, Germany) and 15 patients treated with orthodontic aligners (Invisalign^®^, Align Technology Inc., Santa Clara, CA, USA). The patients were recruited at the Clinical Division of Orthodontics, University Clinic of Dentistry, Medical University of Vienna. The inclusion criteria were age ≥ 18 years, skeletal class I, and minimal mandibular crowding (Little Index grade I–III). The patients with minimal crowding were selected because crowding is a well-known factor facilitating plaque accumulation [17], and we would like to minimize its influence. Exclusion criteria were current smoking, chronic diseases, restorations with close contact to the gingival margin, presence of crowns or bridges, previous non-surgical periodontal treatment (full mouth disinfection, full mouth debridement), medication with antibiotics, steroids, or non-steroidal anti-inflammatory drugs within the past three months, pregnancy, and diabetes. Allocation to treatment modality was based on the patient’s request. 

### 2.3. Saliva Collection

Saliva sampling was conducted during regular patient checkups at three different time points: T0 on the day of treatment beginning but before treatment began, T1 after about three months of treatment, and T2 after about six months. Patients were restricted from eating or drinking (except for water) or chewing gum 1 h before sample collection. This was necessary because eating before the collection might affect the saliva composition and study outcome [18]. Patients had to rinse their mouths directly with 20 mL of distilled water before saliva sampling. Afterward, patients were asked to relax, not swallow their saliva for 2 min, and to spit into a saliva collection beaker (Greiner Bio-One, Kremsmünster, Austria). To ensure that patients collected enough saliva, the bottom of the saliva collection beaker was checked to see if it was fully covered with patient saliva. Collected samples were sterile filtrated (0.22 µm filter, TPP, Trasadingen, Switzerland), aliquoted, and frozen at −80 °C until the application. Saliva collection was only performed from 8 am to 10 am to reduce the influence of circadian rhythm variations of saliva production on the outcome.

### 2.4. Cell Culture

Primary human gingival fibroblasts (hGFs) were obtained from the gingiva around the third molar, extracted from a periodontally healthy individual (female, 18 y.o.) due to orthodontic indications. The donor had no chronic diseases or regular medication intake. Cells were isolated using the outgrowth method [19,20]. The isolated cells were cultured in Dulbecco’s modified Eagle´s medium (DMEM; Sigma Aldrich, St. Louis, MO, USA) supplemented with 10% fetal calf serum (FCS, Gibco, Carlsbad, CA, USA), 100 µg/mL streptomycin (S, Gibco, Carlsbad, CA, USA), and 100 U/mL penicillin (P, Carlsbad, CA, USA) at 37 °C, 5% CO_2_, and 95% humidity. hGFs were passaged after reaching 80% to 90% confluence, and the medium was changed every seven days. Cells from passages 5 and 6 were used for all conducted experiments. Cells from one single donor were used to avoid the contribution of inter-individual variability, which is characteristic of the primary cells [21]. This is because we wanted to focus mainly on the effect of the saliva collected from OT patients.

Human oral squamous carcinoma Ca9-22 cells (Japanese Collection of Research Bioresources Cell Bank, JCRB0625, Ibaraki, Japan) were used as a model for oral epithelial cells. These cells are often used as an oral epithelium model for testing various materials, and although they do not resemble the properties of oral epithelium entirely, their properties remain constant during the propagation in culture conditions [3,22]. Modified Eagle’s minimum essential medium (MEM, Gibco^®^, Carlsbad, CA, USA), supplemented with 10% fetal bovine serum, streptomycin (100 μg/mL), and penicillin (100 U/mL) was used to culture these cells. Ca9-22 cells were cultured at 37 °C in a humidified atmosphere containing 5% CO_2_. For the experiments, cells from passages between 5 and 7 were used.

### 2.5. Stimulation of Cells

One day before stimulation, 5 × 10^4^ hGFs or 1 × 10^5^ Ca9-22 cells were seeded in 24-well plates in 500 µL of the corresponding medium supplemented with FCS and antibiotics P/S. After 24 h of incubation, cells were stimulated with patient saliva diluted in a 1:10 ratio with a corresponding medium supplemented with streptomycin (100 μg/mL) and penicillin (100 U/mL). The dilution was performed because the cells were adapted to grow in a formulated media, and using lower dilutions (e.g., 1:2) would drastically change the composition of the media. Cells incubated in the media without saliva served as a control. Saliva, collected at different time points from the same donor, was applied to the host cells simultaneously to minimize the inter-assay variability. After 4 and 24 h of incubation with saliva, cell viability/proliferation, gene expression of several functional proteins, and the content of IL-8 in the conditioned media were determined. 

### 2.6. Cell Proliferation/Viability

Cell viability/proliferation was assessed using 3-(4,5-Dimethylthiazol-2-yl)-2,5-diphenyltetrazoliumbromid (MTT, Sigma Aldrich, St. Louis, MO, USA) as described in previous studies [3,23]. This method detects the activity of the mitochondria respiratory chain and is considered a measure of the proliferation of viable cells [24]. In brief, at the end of the experiments, 100 µL of MTT solution (5 mg/mL in 1× phosphate-buffered solution, PBS) was added per well, followed by incubation at 37 °C for two hours. After discarding conditioned media, 500 µL of dimethyl sulfoxide (Merck, Darmstadt, Germany) was added per well, followed by 5 min incubation. Finally, 100 µL of the supernatant was transferred in quadruplicates to a 96-well plate, and the optical density (OD) was measured at 570 nm with a photometer (Synergy HTX, Biotek, Winooski, VT, USA).

### 2.7. Gene Expression Analysis

Gene expression levels were quantified using qPCR, similar to our previously published methods [3]. Lysate preparation, mRNA transcription into cDNA, and qPCR were performed using the TaqMan^®^ Gene Expression Cells-to-CT™ kit (Ambion/Applied Biosystems, Foster City, CA, USA) according to the manufacturer’s instructions. Reverse transcription was performed using Primus 96 advanced thermocycler (Applied Biosystems, Foster City, CA, USA) by heating the samples to 37° Celsius for one hour, followed by 95° Celsius for five minutes. qPCR was performed on an ABI StepOnePlus device (Applied Biosystems, Foster City, CA, USA) in paired reactions using TaqMan^®^ gene expression assays. For hGFs, the assays with the following ID numbers (all from Applied Biosystems) were used: interleukin-6 (IL-6, Hs00985639_m1), interleukin-8 (IL-8, Hs00174103_m1), monocyte chemoattractant protein (MCP-1, Hs00234140_m1), and glyceraldehyde 3-phosphate dehydrogenase (GAPDH, Hs99999905_m1). For Ca9-22, the following TaqMan gene expression assays were used: IL-6, IL-8, integrin α6 (ITGα6, Hs01041011_m1), integrin α5 (ITGα5, Hs01547673_m1), integrin β4 (ITGβ4, Hs00173995_m1), E-cadherin, (Hs01023894_m1), and GAPDH. The PCR reactions were performed in duplicates under the following conditions: 95 °C for 10 min, followed by 50 cycles each of 15 s at 95 °C and 1 min at 60 °C. For each sample, the point at which the PCR product was first detected above a fixed threshold was determined as the cycle threshold (*Ct*). The 2^−ΔΔCt^ method, which is a standard method for qPCR data analysis [25], was used to calculate the relative expression of target genes using GAPDH as a housekeeping gene and unstimulated cells as a control with the following formula:

$${{∆∆C}_{t}=(C_{t}}^{target}-{C_{t}}^{GAPDH}{)}_{sample}-({C_{t}}^{target}-{C_{t}}^{GAPDH}{)}_{control}$$

### 2.8. ELISA

The conditioned media were harvested at the end of the experiment, centrifuged, and frozen at −80 °C until the analysis. Protein levels of IL-8 were measured in the supernatant collected at the end of the stimulation of Ca9-22 cells using Human Uncoated IL-8 ELISA (from Thermo Fisher Scientific, Waltham, MA, USA) according to the manufacturer’s instructions. OD was measured at 450 nm and 570 nm using a Synergy HTX multi-mode reader (BioTek Instruments, Winooski, VT, USA). After background subtraction and subtracting OD_570_ from OD_450_, final concentrations were calculated by plotting determined OD values against the appropriate standard curve using Gen5 All-In-One Microplate Reader Software version 2.09 (BioTek Instruments, Winooski, VT, USA). The detection limit of IL-8 ELISA was 2 pg/mL; all values below this limit were considered as zero for the analysis.

### 2.9. Statistical Analysis

Differences between the Invisalign and bracket groups were analyzed with the Mann–Whitney test. Differences between various time points were analyzed by the Wilcoxon test. The non-parametric tests were used because, with the sample size in our study (*n* < 16), it is impossible to reach a conclusion about the normal distribution and, therefore, the application of parametric tests was not justified. Statistically significant differences were confirmed if *p*-values were less than 0.05. Statistical analysis was performed using SPSS 26.0 software (IBM, Armonk, NY, USA). Data are presented as mean ± s.e.m. Each experiment was performed in technical duplicates.

## 3. Results

This prospective study included 15 Invisalign patients (seven females and eight males, mean age 28.2 ± 1.8) and 16 bracket patients (six females and ten males, mean age 29.1 ± 2.2). No difference in age between the two groups was observed.

### 3.1. Proliferation/Viability of hGFs and Ca9-22 Cells

**Figure 1:**
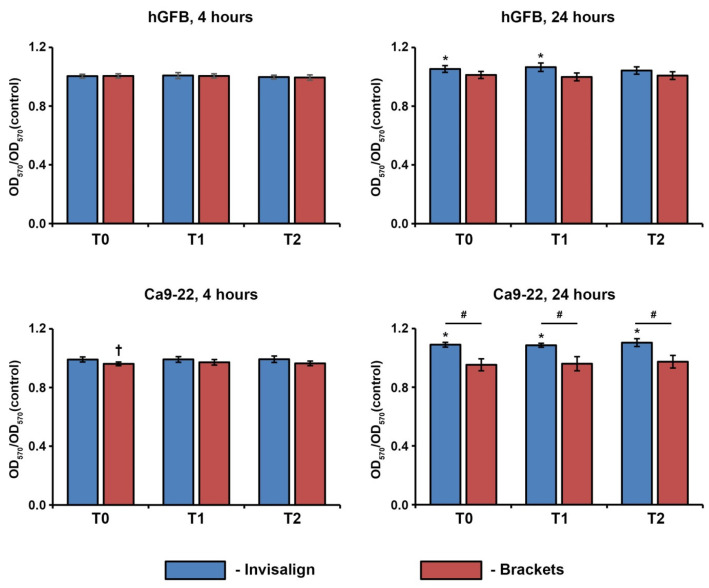
Effect of the saliva of the patients undergoing OT on the proliferation/viability of hGFs and Ca9-22 cells. *—significantly higher compared to the untreated controls, *p* < 0.05. †—significantly lower compared to the untreated controls, *p* < 0.05. #—significantly different between Invisalign and brackets.

Figure 1 shows the viability of hGFs and Ca9-22 cells treated with the saliva of Invisalign and brackets patients for 4 and 24 h in relation to that of untreated cells. The saliva of Invisalign patients collected at T0 and T1 slightly stimulated the viability of hGFs after 24 h stimulation. No difference between the viability of hGFs treated with the saliva of Invisalign and bracket patients was observed. In Ca-9-22 cells, treatment with the saliva of Invisalign patients for 24 h resulted in significantly higher viability than the untreated controls and cells treated with the saliva of brackets patients collected at all time points.

Cells were treated for 4 or 24 h with the saliva of the patients undergoing OT with either Invisalign or brackets collected immediately before (T0), about three months after (T1), and about six months (T2) after beginning the orthodontic treatments. The optical densities (OD) were measured at 570 nm and normalized to those measured for untreated controls. Data are presented as means ± s.e.m. of 15 Invisalign patients and 16 bracket patients.

### 3.2. Gene Expression of IL-6, IL-8, MCP-1 in hGFs

**Figure 2:**
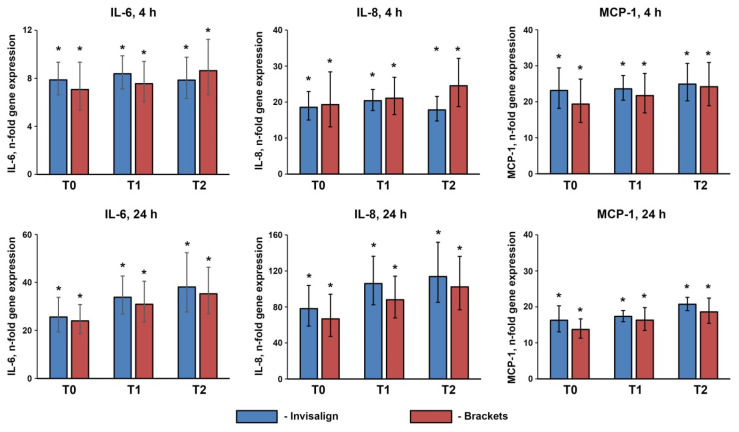
Effect of saliva of the patients undergoing OT on the gene expression of proinflammatory mediators in hGFs. *—significantly higher compared to the untreated controls, *p* < 0.05.

Figure 2 shows the gene expression levels of the proinflammatory mediators IL-6, IL-8, and MCP-1. All of the analyzed genes were expressed significantly higher (*p* < 0.005) in hGFs treated with patient saliva compared to cell culture medium after 4 and 24 h of incubation. There was no significant difference between the saliva of Invisalign^®^ and bracket patients regarding gene expression levels of hGFs (*p* > 0.6). Furthermore, the increased expression of proinflammatory markers was not dependent on the time point during OT. Since no difference in the gene expression of these mediators was detected between different OTs and time points, no protein analysis was performed. 

Primary hGFs were treated for 4 or 24 h with the saliva of the patients undergoing OT with either Invisalign or brackets collected immediately before (T0), about three months after (T1), and about six months (T2) after beginning the orthodontic treatments. The resulting gene expression of IL-6, IL-8, and MCP-1 was measured by qPCR. Y-axes show *n*-fold gene expression calculated using the 2^−ΔΔCt^ method in relation to the untreated controls. Data are presented as means ± s.e.m. of 15 Invisalign patients and 16 bracket patients.

### 3.3. IL-8 Gene Expression and Protein Production by Ca9-22 Cells

**Figure 3:**
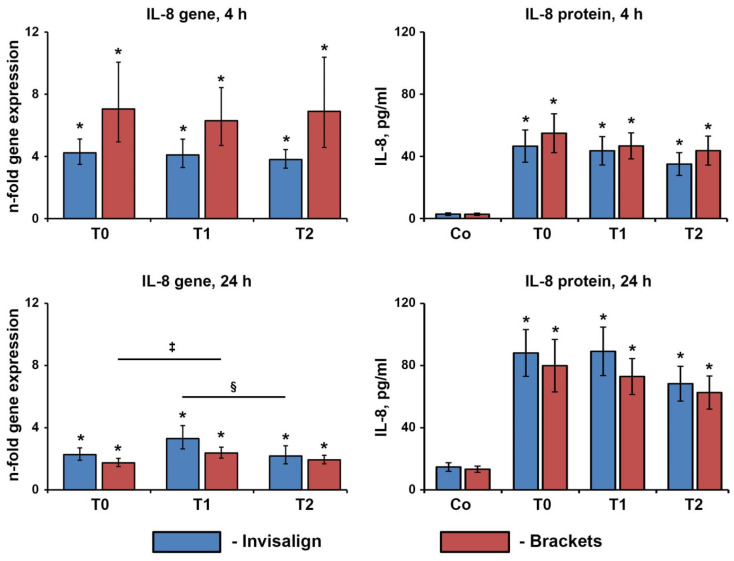
Effect of saliva of the patients undergoing OT on the gene expression and protein production of IL-8 by Ca9-22 cells. *—significantly higher compared to the untreated controls, *p* < 0.05. ‡—significantly different between T0 and T1, *p* < 0.05. §—significantly different between T1 and T2.

The gene expression and protein production of IL-8 after treatment of Ca9-22 cells with the saliva of the patients undergoing OT is shown in Figure 3. After 4 h of stimulation, the saliva of both Invisalign and bracket patients induced a significant increase in IL-8 gene expression and protein production. No significant difference was observed between the two OTs, but a slightly higher response was observed for the bracket patients (*p* > 0.16). Increased gene and protein levels of IL-8 in Ca9-22 cells were also observed after 24 h of stimulation with the saliva of OT patients. The saliva collected three months after therapy began induced higher IL-8 gene expression levels than that collected before the therapy; significant differences were observed for the Invisalign group. The saliva collected in six months induced lower IL-8 gene expression levels than those collected in three months; statistically significant differences were observed for bracket patients. 

Ca9-22 cells were treated for 4 or 24 h with the saliva of patients undergoing OT with either Invisalign or brackets collected immediately before (T0), about three months after (T1), and about six months (T2) after beginning the orthodontic treatments. The resulting gene expression (left panels) and protein production (right panels) of IL-8 were measured by qPCR and ELISA, respectively. Y-axes in the left panels show *n*-fold gene expression calculated by the 2^−ΔΔCt^ method in relation to the untreated controls (*n*-fold expression = 1). Y-axes in the right panels show the concentration of IL-8 in the conditioned media at the end of the treatment. Data are presented as means ± s.e.m. of 15 Invisalign patients and 16 bracket patients.

### 3.4. Gene Expression of E-Cadherin, ITGα6, and ITGβ4 in Ca9-22

**Figure 4:**
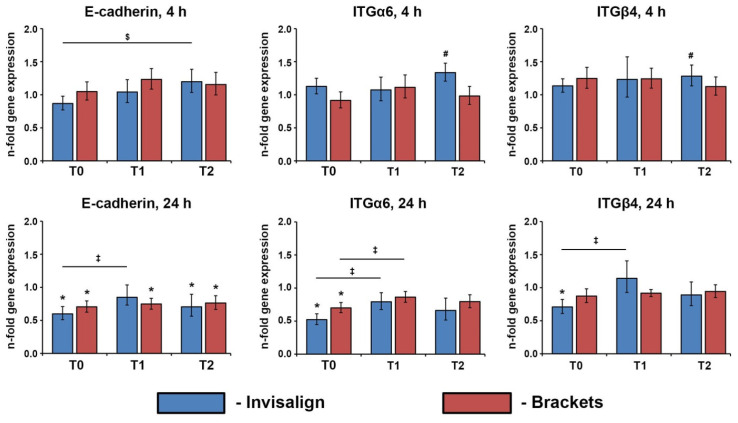
Effect of saliva of the patients undergoing OT on the gene expression of E-cadherin, ITGα6, and ITGβ4 in Ca9-22 cells. *—significantly lower compared to the untreated controls, *p* < 0.05. #—significantly higher compared to the untreated control, *p* < 0.05. ‡—significantly different between T0 and T1, *p* < 0.05. $—significantly different between T0 and T2, *p* < 0.05.

The effect of the saliva of OT patients on the expression of E-cadherin, ITGα6, and ITGβ4 is shown in Figure 4. The gene expression of E-cadherin in Ca9-22 was slightly but significantly decreased after 24 h of stimulation with the saliva of the patients undergoing OT in all cases except Invisalign patients at T1. The resulting gene expression of E-cadherin after stimulation with the saliva of Invisalign patients was slightly increased in the course of the therapy; significant changes were observed between time points T2 and T0 after 4 h stimulation and between T1 and T0 after 24 h stimulation. The gene expression of ITGα6 in Ca9-22 cells was significantly decreased upon stimulation with the saliva of OT patients collected before the therapy (T0) for 24 h. The saliva collected three months after OT began induced significantly higher ITGα6 levels compared to the saliva collected before the therapy; a significant effect was observed for both Invisalign and brackets after 24 h stimulation. The gene expression of ITGβ4 was almost unaffected by the saliva of OT patients, excepting a significant decrease for Invisalign patients at T0 after 24 h stimulation and a significant increase for Invisalign patients at T2 after 4 h stimulation. 

Ca9-22 cells were treated for 4 or 24 h with the saliva of the patients undergoing OT with either Invisalign or brackets collected immediately before (T0), about three months after (T1), and about six months (T2) after beginning the orthodontic treatments. The resulting gene expression of E-cadherin, ITGα6, and ITGβ4 were measured by qPCR. Y-axes show *n*-fold gene expression calculated by the 2^−ΔΔCt^ method in relation to the untreated controls (*n*-fold expression = 1). Data are presented as means ± s.e.m. of 15 Invisalign patients and 16 bracket patients.

## 4. Discussion

Orthodontic treatment is known to be accompanied by the transient worsening of oral health due to impaired ability to perform adequate oral hygiene. However, it is unclear if there are some differences between various OT types, particularly brackets vs. aligners. Saliva is considered to be an important instrument for oral health monitoring [9,10]. Therefore, in the present study, we used the saliva collected before and during OT from patients treated with Invisalign and brackets for the stimulation of human gingival fibroblasts and oral epithelial cells in vitro, aiming to find any difference between different OT protocols. 

The saliva of orthodontic patients had no toxic effect on the proliferation/viability of hGFs and Ca9-22 cells assessed by the MTT method. This method is based on the ability of cell mitochondria to convert the MTT reagent into formazan and is an indicator of cell viability and proliferation [15,24]. Our finding is in agreement with previous studies [15,16,26]. No differences in the proliferation/viability were found upon the stimulation of hGFs with the saliva of the bracket and Invisalign patients. However, in Ca9-22 cells, stimulation with the saliva of Invisalign patients resulted in significantly higher proliferation/viability than stimulation with the saliva of bracket patients. This was observed at all time points, even before the therapy began, and, therefore, could not be attributed to the different OTs but instead to the various intrinsic properties of the saliva in the patients of Invisalign and bracket groups. 

Human gingival fibroblasts are the major cells in the connective tissue and participate in tissue remodeling, the inflammatory response, and immunomodulation [27,28]. We showed that the saliva of patients undergoing OT induced a significant increase in the gene expression of IL-6, IL-8, and MCP-1. However, no significant difference between the bracket and Invisalign groups was observed at any saliva collection point and after both 4 and 24 h of stimulation. Therefore, it can be concluded that the type of OT and OT itself do not influence the ability of saliva to induce the inflammatory response in hGFs. The ability of the saliva to induce the production of proinflammatory factors in hGFs is also shown by previous studies [15,29], but its physiological role is unclear. 

We have further investigated the effect of the saliva of OT patients on Ca9-22 cells. These cells are often used as a model of oral epithelium, even if they do not entirely reflect all its properties. Oral epithelium fulfills immunological and barrier functions in protecting the oral cavity from invading microorganisms [30,31]. IL-8 production by the oral epithelium is crucial for recruiting neutrophils [32], which play a major role in pathogen elimination [33]. We found that the saliva of OT patients stimulated the production of IL-8 by Ca9-22 cells, but no difference was observed between brackets and aligners. Interestingly, a recent study also showed that saliva can induce IL-8 secretion by the GMSM-K epithelial cell line [16]. In contrast, artificial saliva does not induce IL-8 gene expression in oral squamous carcinoma HSC-2 cells [26]. The ability of saliva to induce IL-8 production could be physiologically crucial because it may ensure the continuous migration of neutrophils into the oral cavity and, thus, effective antibacterial defense. A tendency can be noted that saliva collected at T2 induced lower levels of IL-8 than that collected at T1. This observation, although in most cases not statistically significant, may suggest that any potential detrimental effect of OT is more pronounced in the early phase than in the later phase; specific studies with higher participant numbers should further approve this. 

Besides the inflammatory role, oral epithelial cells also exert an important role in barrier function [30]. This function is partially mediated by E-cadherin, which is involved in the intercellular contacts between adjacent cells [34], and the integrin subunits ITGα6 and ITGβ4, which are involved in the adhesion of epithelial cells to the basal membrane [35]. We found that the saliva of the study participants induced a significant decrease in the gene expression of E-cadherin, ITGα6, and ITGβ4, suggesting that the saliva might affect the barrier function of oral epithelium. The assumption that saliva might regulate the barrier function of the oral epithelium is also confirmed by the recent finding of Roy et al., who reported an increase in the transepithelial electrical resistance of cells treated with saliva, which was attributed to the presence of some growth factors [16]. Interestingly, the saliva collected three months after the therapy induced slightly higher gene expression levels of barrier proteins, suggesting that OT might slightly change the properties of saliva. Similarly to other parameters, we did not observe any difference between the saliva of Invisalign and bracket patients in the ability to affect the gene expression of the barrier proteins E-cadherin, ITGα6, and ITGβ4.

In order to minimize the effect of inter-assay variability on the study outcome, we used the saliva collected at the different treatment points within the same experiment on host cells. To achieve this, the saliva was immediately aliquoted and stored at −80°C after collection. A previous study suggested that the storage of saliva at −80°C preserves all its components [36,37]. Of note, a similar protocol of freezing the saliva at −80 °C was also applied in a recent study with periodontitis patients [16]. 

The data of our study do not favor either clear aligners or brackets regarding the inflammatory status and the ability of saliva to induce the inflammatory response in host cells. Moreover, no changes in the investigated parameters were observed in the course of the therapy. These data contradict some previous studies. Particularly, a recent clinical study showed an increase in the levels of several inflammatory markers in the gingival crevicular fluid of orthodontic patients treated with either aligners or fixed brackets one month after the beginning of the therapy [38]. This study also found some differences between aligners and brackets, but no clear tendency was found: some parameters, particularly interleukin (IL)-1a, IL-2, IL-6, and IL-8, were higher in aligner patients, whereas others like TNF-a and RANKL were increased in bracket patients [38]. Another study also reported an increase in several inflammatory markers in the gingival crevicular fluid of orthodontic patients after three weeks of treatment, with no difference between aligners and fixed appliances [39]. Some differences between our study and the literature data could be explained by different biological materials and time points after the therapy. 

We did not find any essential alteration in the ability of the saliva to stimulate host cells during the orthodontic therapy, nor any difference between the patients treated with brackets or aligners. This finding does not mean that OT has no detrimental effect on saliva properties and composition. Our study focused only on one of the aspects of saliva properties, but further facets, like the impact of OT on salivary proteomics and microbiology, still need to be investigated. Furthermore, the role of appropriate oral hygiene instructions and their following by the patients on the study parameters could be essential. 

Our study has some limitations, particularly a low number of participants and in vitro character. Furthermore, as mentioned above, Ca9-22 is a squamous carcinoma cell line that does not reflect the oral epithelium entirely. We diluted saliva before application to the cells, which was unavoidable because the cells required culture media components for survival. 

## 5. Conclusions

In summary, in the present study, we showed that different OT modes, namely Invisalign and bracket treatments, do not affect the effect of their saliva on human gingival fibroblasts and oral epithelial cells. Furthermore, the differences in the saliva collected before and after therapy began were minimal. These findings contradict the data of clinical studies, showing an increase in salivary levels of proinflammatory markers in the course of OT. These differences are due to different study designs and could also be due to the additional processing of saliva in our study. Nevertheless, the ability of saliva to influence the inflammatory reactions and barrier properties of gingival fibroblasts and the oral epithelium is an interesting phenomenon, and its physiological importance is still to be understood.

## Data Availability

The data presented in this study are available on request from the corresponding author.

## References

[1] Vlaskalic V., Boyd R. (2001). Orthodontic treatment of a mildly crowded malocclusion using the Invisalign System. Aust. Orthod. J..

[2] Christou T., Abarca R., Christou V., Kau C.H. (2020). Smile outcome comparison of Invisalign and traditional fixed-appliance treatment: A case-control study. Am. J. Orthod. Dentofac. Orthop..

[3] Nemec M., Bartholomaeus H.M., Michael H.B., Behm C., Ali Shokoohi-Tabrizi H., Jonke E., Andrukhov O., Rausch-Fan X. (2020). Behaviour of Human Oral Epithelial Cells Grown on Invisalign^®^ SmartTrack^®^ Material. Materials.

[4] Lu H., Tang H., Zhou T., Kang N. (2018). Assessment of the periodontal health status in patients undergoing orthodontic treatment with fixed appliances and Invisalign system: A meta-analysis. Medicine.

[5] Jiang Q., Li J., Mei L., Du J., Levrini L., Abbate G.M., Li H. (2018). Periodontal health during orthodontic treatment with clear aligners and fixed appliances: A meta-analysis. J. Am. Dent. Assoc..

[6] ElNaghy R., Al-Qawasmi R., Hasanin M. (2023). Does orthodontic treatment using clear aligners and fixed appliances affect periodontal status differently?. Evid. -Based Dent..

[7] Di Spirito F., D’Ambrosio F., Cannata D., D’Anto V., Giordano F., Martina S. (2023). Impact of Clear Aligners versus Fixed Appliances on Periodontal Status of Patients Undergoing Orthodontic Treatment: A Systematic Review of Systematic Reviews. Healthcare.

[8] Giannobile W.V., Beikler T., Kinney J.S., Ramseier C.A., Morelli T., Wong D.T. (2009). Saliva as a diagnostic tool for periodontal disease: Current state and future directions. Periodontology.

[9] Haririan H., Andrukhov O., Laky M., Rausch-Fan X. (2021). Saliva as a Source of Biomarkers for Periodontitis and Periimplantitis. Front. Dent. Med..

[10] Paqué P.N., Hjerppe J., Zuercher A.N., Jung R.E., Joda T. (2022). Salivary biomarkers as key to monitor personalized oral healthcare and precision dentistry: A scoping review. Front. Oral Health.

[11] Jaedicke K.M., Preshaw P.M., Taylor J.J. (2016). Salivary cytokines as biomarkers of periodontal diseases. Periodontology.

[12] Syndergaard B., Al-Sabbagh M., Kryscio R.J., Xi J., Ding X., Ebersole J.L., Miller C.S. (2014). Salivary biomarkers associated with gingivitis and response to therapy. J. Periodontol..

[13] Kc S., Wang X.Z., Gallagher J.E. (2020). Diagnostic sensitivity and specificity of host-derived salivary biomarkers in periodontal disease amongst adults: Systematic review. J. Clin. Periodontol..

[14] Pourgonabadi S., Muller H.D., Mendes J.R., Gruber R. (2017). Saliva initiates the formation of pro-inflammatory macrophages in vitro. Arch. Oral Biol..

[15] Cvikl B., Lussi A., Moritz A., Sculean A., Gruber R. (2015). Sterile-filtered saliva is a strong inducer of IL-6 and IL-8 in oral fibroblasts. Clin. Oral Investig..

[16] Roy A., Ben Lagha A., Goncalves R., Grenier D. (2021). Effects of Saliva From Periodontally Healthy and Diseased Subjects on Barrier Function and the Inflammatory Response in in vitro Models of the Oral Epithelium. Front. Oral Health.

[17] Alsulaiman A.A., Kaye E., Jones J., Cabral H., Leone C., Will L., Garcia R. (2018). Incisor malalignment and the risk of periodontal disease progression. Am. J. Orthod. Dentofac. Orthop..

[18] Hanrahan K., McCarthy A.M., Kleiber C., Lutgendorf S., Tsalikian E. (2006). Strategies for salivary cortisol collection and analysis in research with children. Appl. Nurs. Res. ANR.

[19] Blufstein A., Behm C., Kubin B., Gahn J., Moritz A., Rausch-Fan X., Andrukhov O. (2022). Anti-apoptotic effects of human gingival mesenchymal stromal cells on polymorphonuclear leucocytes. Oral Dis..

[20] Andrukhov O., Behm C., Blufstein A., Wehner C., Gahn J., Pippenger B., Wagner R., Rausch-Fan X. (2020). Effect of implant surface material and roughness to the susceptibility of primary gingival fibroblasts to inflammatory stimuli. Dent. Mater..

[21] Chansard A., Dubrulle N., Poujol de Molliens M., Falanga P.B., Stephen T., Hasan M., van Zandbergen G., Aulner N., Shorte S.L., David-Watine B. (2020). Unveiling Interindividual Variability of Human Fibroblast Innate Immune Response Using Robust Cell-Based Protocols. Front. Immunol..

[22] Garcia-Contreras R., Scougall-Vilchis R.J., Contreras-Bulnes R., Kanda Y., Nakajima H., Sakagami H. (2014). Cytotoxicity and pro-inflammatory action of chemo-mechanical caries-removal agents against oral cells. Vivo.

[23] An N., Holl J., Wang X., Rausch M.A., Andrukhov O., Rausch-Fan X. (2021). Potential Suppressive Effect of Nicotine on the Inflammatory Response in Oral Epithelial Cells: An In Vitro Study. Int. J. Environ. Res. Public Health.

[24] Mosmann T. (1983). Rapid colorimetric assay for cellular growth and survival: Application to proliferation and cytotoxicity assays. J. Immunol. Methods.

[25] Rao X., Huang X., Zhou Z., Lin X. (2013). An improvement of the 2^−ΔΔCT^ method for quantitative real-time polymerase chain reaction data analysis. Biostat. Bioinform. Biomath..

[26] Muller H.D., Cvikl B., Lussi A., Gruber R. (2016). Chemokine expression of oral fibroblasts and epithelial cells in response to artificial saliva. Clin. Oral Investig..

[27] Andrukhov O., Behm C., Blufstein A., Rausch-Fan X. (2019). Immunomodulatory properties of dental tissue-derived mesenchymal stem cells: Implication in disease and tissue regeneration. World J. Stem Cells.

[28] Alfonso Garcia S.L., Parada-Sanchez M.T., Arboleda Toro D. (2020). The phenotype of gingival fibroblasts and their potential use in advanced therapies. Eur. J. Cell Biol..

[29] Muller H.D., Cvikl B.B., Lussi A.A., Gruber R.R. (2016). Salivary pellets induce a pro-inflammatory response involving the TLR4-NF-kB pathway in gingival fibroblasts. BMC Oral Health.

[30] Groeger S., Meyle J. (2019). Oral Mucosal Epithelial Cells. Front. Immunol..

[31] Moutsopoulos N.M., Konkel J.E. (2018). Tissue-Specific Immunity at the Oral Mucosal Barrier. Trends Immunol..

[32] Tonetti M.S., Imboden M.A., Lang N.P. (1998). Neutrophil migration into the gingival sulcus is associated with transepithelial gradients of interleukin-8 and ICAM-1. J. Periodontol..

[33] Uriarte S.M., Edmisson J.S., Jimenez-Flores E. (2016). Human neutrophils and oral microbiota: A constant tug-of-war between a harmonious and a discordant coexistence. Immunol. Rev..

[34] Abe-Yutori M., Chikazawa T., Shibasaki K., Murakami S. (2017). Decreased expression of E-cadherin by Porphyromonas gingivalis-lipopolysaccharide attenuates epithelial barrier function. J. Periodontal Res..

[35] Sonnenberg A., Calafat J., Janssen H., Daams H., van der Raaij-Helmer L.M., Falcioni R., Kennel S.J., Aplin J.D., Baker J., Loizidou M. (1991). Integrin alpha 6/beta 4 complex is located in hemidesmosomes, suggesting a major role in epidermal cell-basement membrane adhesion. J. Cell Biol..

[36] Karched M., Bhardwaj R.G., Pauline E.M., George S., Asikainen S. (2017). Effect of preparation method and storage period on the stability of saliva DNA. Arch. Oral Biol..

[37] Chevalier F., Hirtz C., Chay S., Cuisinier F., Sommerer N., Rossignol M., de Périère D.D. (2007). Proteomic Studies of Saliva: A Proposal for a Standardized Handling of Clinical Samples. Clin. Proteom..

[38] Kamran M.A., Alnazeh A.A., Almagbol M., Almoammar S., Alhaizaey A.H.A., Alshahrani I. (2023). Role of six cytokines and bone metabolism biomarkers in gingival crevicular fluid in patients undergoing fixed orthodontic appliance treatment in comparison with aligners: A clinical study. Angle Orthod..

[39] Gujar A.N., Baeshen H.A., Alhazmi A., Bhandi S., Raj A.T., Patil S., Birkhed D. (2019). Cytokine levels in gingival crevicular fluid during orthodontic treatment with aligners compared to conventional labial fixed appliances: A 3-week clinical study. Acta Odontol. Scand..

